# Transmitarray-Based CATR for Streamline UAV RCS Measurement

**DOI:** 10.3390/s26051455

**Published:** 2026-02-26

**Authors:** Jiazhi Tang, Run-Kuan Liu, Xiaoming Chen, Guan-Long Huang, Xiuyin Zhang

**Affiliations:** 1 School of Electronic and Information Engineering, South China University of Technology, Guangzhou 510641, China; 2 School of Electronic and Information Engineering, Foshan University, Foshan 528225, China; 3 School of Computer Science and Artificial Intelligence, Foshan University, Foshan 528225, China; 4 School of Information and Communications Engineering, Xi’an Jiaotong University, Xi’an 710049, China; 5 School of Microelectronics, South China University of Technology, Guangzhou 510640, China

**Keywords:** radar cross section (RCS), streamline measurement, transmitarray compact antenna test range (TA-CATR)

## Abstract

Low-altitude unmanned communication platforms play a vital role in integrated sensing and communication (ISAC) systems. Due to the large number of deployed devices and their high level of functional integration, there are high demands for rapid and effective over-the-air testing. However, conventional measurement systems are limited by structural complexity and size, making them unsuitable for compact, efficient, and mass-production testing. To address these challenges, this paper proposes a rapid radar cross section (RCS) measurement method for streamline measurement of batch low-altitude communication equipment, based on a transmitarray compact antenna test range (TA-CATR). The proposed method performs RCS measurements over elevation and azimuth angles by combining background subtraction, extrapolation, and interpolation techniques, achieving measurable ranges of −45° to 45° and 0° to 360°, respectively. This approach offers a compact, cost-effective, and highly efficient solution for large-scale production testing. Experimental results validate the accuracy and practicality of the proposed TA-CATR-based RCS measurement method.

## 1. Introduction

Radar cross section (RCS) measurement is a fundamental tool for assessing electromagnetic scattering behavior and, consequently, target detectability in practical sensing and radar systems [1,2,3,4,5,6,7,8,9]. It is widely used not only for signature evaluation and comparison across different platforms, but also for validating electromagnetic models, quantifying the influence of structural details, and supporting engineering verification during design, integration, and acceptance testing. In many applications, reliable RCS characterization is required over a range of observation angles and frequencies, and the measurement results must maintain adequate repeatability under realistic test conditions. However, traditional far-field RCS characterization relies on strict far-field distance requirements, where the separation between the radar antennas and the target must be sufficiently large to approximate plane-wave illumination and stable angular resolution. For electrically large targets, this requirement translates into extremely large test ranges, which significantly increases facility footprint and cost, and can introduce additional challenges such as alignment complexity, environmental clutter, and reduced measurement efficiency. These limitations become even more pronounced for millimeter-wave systems, where higher frequencies impose tighter tolerances on positioning, stability, and instrumentation, and where large-scale ranges are harder to realize with acceptable uncertainty and measurement efficiency.

To mitigate these constraints while preserving far-field equivalence, compact antenna test ranges (CATRs) have been developed to generate a controlled quiet zone that approximates plane-wave illumination within a restricted space. By employing a collimating structure, such as a shaped reflector or a transmissive aperture, a CATR can transform the spherical wavefront from a feed into a quasi-planar wavefront over a designated region, enabling far-field-like RCS measurements without requiring the full far-field distance. This approach substantially reduces the required facility size and improves practicality for laboratory and industrial environments, while also offering enhanced measurement efficiency for repeated tests across multiple targets or configurations [10,11,12].

Compact antenna test ranges can be realized in several representative forms [11,12,13,14,15,16,17,18,19]. Reflector-based CATRs employ a parabolic or shaped reflector, in single-reflector or dual-reflector arrangements, to collimate the spherical wave from a feed into a quasi-plane wave within a quiet zone, and edge treatments are often introduced to suppress diffraction and improve field uniformity. Lens-based CATRs use dielectric or quasi optical lenses, such as dielectric lenses, Fresnel lenses, or gradient index lenses, to achieve wavefront collimation with low blockage and potentially wideband operation, while being constrained by material loss and fabrication tolerances at high frequencies. More recently, transmitarray or metasurface based CATRs have attracted increasing attention because a planar transmissive aperture can impose a prescribed spatial phase, and sometimes amplitude, distribution to synthesize a near planar wavefront in a compact volume, with passive implementations offering stable fixed collimation and reconfigurable implementations enabling adjustable phase distributions for enhanced flexibility [12]. In addition, cylindrical CATRs collimate the field in one dimension to form a line-type quiet zone for specialized measurements. For RCS applications, these CATR collimators can be combined with monostatic or bistatic measurement geometries, depending on whether a shared transmit receive path or separated antennas are preferred for the intended test setup.

A particularly relevant scenario is streamline RCS testing of autonomous aerial vehicles (AAVs) or unmanned aerial vehicles (UAVs). Here, UAV refers to generic unmanned aircraft, while AAV emphasizes autonomy and intelligent operation, which are crucial for future 6G and integrated sensing-and-communication (ISAC) applications [20,21,22,23,24,25]. With the rapid proliferation of UAV/AAV platforms in surveillance, transportation, and communication networks, cost-effective and repeatable scattering evaluations are attracting increasing attention. Conventional large-scale anechoic chamber or CATR facilities are unsuitable for mass production testing due to their size, complexity, and cost. Thus, a type of transmitarray compact antenna test range (TA-CATR) offers a compact, modular, and lightweight platform that can be deployed on production lines [26,27]. Its ability to provide uniform quiet zones makes it highly suitable for UAV/AAV RCS verification and mass-production device evaluation. Despite these advantages, the currently designed TA-CATR operates in a near-field environment and features a much more compact setup compared with the conventional CATR [23]. In this case, the mutual coupling between the transmitting and receiving feed antennas as well as the residual scattered fields exist. If these effects are not properly accounted for during the measurement, the resulting data will be distorted. Addressing these effects is therefore essential to ensure accurate and reliable RCS characterization in the proposed system.

For RCS characterization, the quiet zone quality is decisive because the target must be illuminated by a field that is as close as possible to a uniform plane wave. Any amplitude ripple across the quiet zone changes the effective weighting of different parts of the target, while phase distortion introduces wavefront curvature and angular errors. Both effects can bias the extracted RCS and increase measurement uncertainty, especially when the target is electrically large or when wide angular coverage is required. In practical compact ranges, the measurement is further degraded by unwanted reflections and multipath contributions originating from the chamber walls, absorbers with finite performance, cable routing, and supporting fixtures such as mounts, positioners, and brackets. These parasitic scattering components may be comparable to the device under test (DUT) response at certain angles and frequencies, thereby contaminating the measured scattering signature.

To mitigate these issues, the proposed TA-CATR integrates a background subtraction procedure that is widely adopted in conventional RCS measurements. In this workflow, the background scene, a calibration object with a known scattering response, and the device under test are measured sequentially under identical settings. The recorded responses are then processed to suppress quasi-static clutter and coupling terms, and to normalize the measurement using the calibration object, yielding calibrated RCS results with improved repeatability. This integrated framework, which was deliberately conceived in advance with production-line verification as a primary target scenario, is particularly suitable for industrial deployment. Conventional far-field ranges are often impractical in such settings because of strict space requirements, high facility and maintenance cost, and limited measurement efficiency. By contrast, the proposed workflow is designed to support fast, consistent, and automation-friendly measurements under compact site constraints, enabling repeatable RCS characterization for batch inspection and routine acceptance testing. In this work, the measurement platform adopts the transmitarray-based CATR reported in our previous work [12], which has been experimentally validated and is cited in this manuscript. The adopted CATR provides a low-profile and lightweight solution while maintaining a sizeable quiet zone with controlled field errors, thereby enabling a compact measurement cell suitable for production line deployment. The referenced platform achieves a very small F/D of 0.287 and a quiet zone aperture ratio of about 50%, and it has been characterized over 2.4–2.75 GHz (13.5% fractional bandwidth), which supports the compact and practical RCS measurements targeted in this study.

In this paper, the design, simulation, and experimental validation of a TA-CATR dedicated to streamline RCS measurement are reported. Based on the TA-CATR, a new testing technique including background subtraction, extrapolation, and interpolation is applied for the RCS measurement. Numerical and experimental results confirm the effectiveness of the proposed approach for bistatic RCS testing. Compared with conventional reflector-based CATRs, the proposed system achieves compact form factor, simplified fabrication, and strong adaptability to RCS scenarios. The main contribution is a TA-CATR measurement framework for production-line deployment that integrates compact hardware and lightweight processing to achieve repeatable wide-angle RCS acquisition under severe space constraints.

## 2. RCS Streamline Measurement

**Figure 1:**
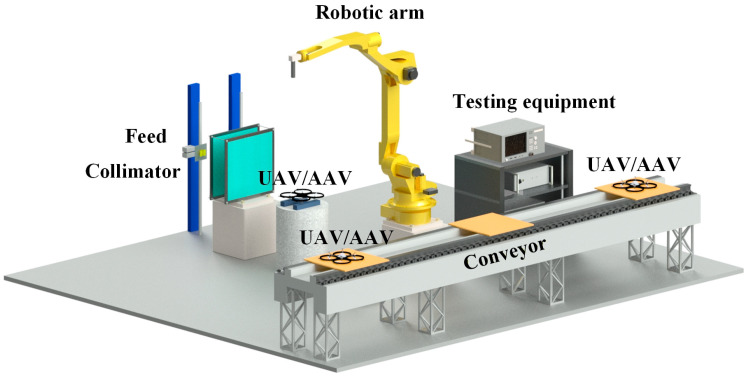
Schematic of a streamline UAV/AAV bistatic RCS measurement system.

To enable accurate RCS extraction under such an open industrial environment, Figure 1 presents an automated streamline testing scenario designed for UAV/AAV equipment. Since such unmanned platforms are lightweight and produced in large quantities, a conveyor inspection setup is adopted to enable rapid factory testing. As shown in Figure 1, the high efficiency testing environment consists of a mechanical conveyor, industrial robotic arms, testing instruments, a transmitarray collimator and two antennas. The transmitarray-based compact antenna test range in this work is implemented as a compact collimation module composed of a feed antenna and two cascaded transmissive metasurfaces, namely an amplitude modification metasurface and a phase modulation metasurface. The two metasurfaces share the same unit cell topology but are configured with different element selections to realize amplitude shaping and phase compensation, respectively, so that a quasi planar wavefront can be synthesized within a designated quiet zone. The prototype operates around 2.6 GHz with a relative working frequency bandwidth of 13.5%. The metasurface aperture is 501.7 mm × 501.7 mm with an overall thickness of 14.572 mm. A short focal length of 144 mm is adopted, corresponding to an F/D of 0.287, to reduce the range length while maintaining a usable quiet zone. The aperture ratio of the CATR is 50%. The quiet zone uniformity of the TA CATR platform was experimentally quantified in our referenced work [12] by near field scanning on the quiet zone plane, and it meets the above criterion at 2.6 GHz. Compared with conventional reflector-based CATRs, the proposed transmitarray collimator provides an inherent polarization conversion capability and supports a small F/D configuration. These features help suppress edge-related diffraction and improve the quiet-zone field quality, so additional edge treatments such as serrated rims or blended edges are not required. During the industrial inspection process, the UAVs or AAVs under test are conveyed at a controlled speed into the designated test region. The robotic arm positions the device within the quiet zone area of the TA-CATR, where the CATR system automatically performs RCS and performance measurements. A stop and measure workflow is used for repeatability: acquisition is triggered only after the DUT is stationary. Background subtraction and periodic reference target monitoring are employed to mitigate environmental reflections and drift in industrial environments.

In the proposed streamline inspection environment, the TA-CATR is intended to operate in an open industrial setting that typically contains multiple metallic structures, conveyor mechanisms, and robotic manipulators for automated handling and positioning. Such a deployment scenario is fundamentally different from a traditional anechoic chamber. The primary design objective is to achieve compact footprint, high measurement efficiency, and seamless integration with automation equipment, rather than to provide complete electromagnetic isolation. As a result, the electromagnetic scene inevitably includes many nearby scatterers, including mechanical supports, mounting frames, conveyor surfaces, cable trays, and surrounding fixtures, as well as transient scattering from machine during operation. These parasitic scattering and multipath contributions can couple into the receive channel and superimpose on the target response, leading to measurement bias if they are not properly suppressed. Therefore, robust clutter mitigation and repeatable calibration become essential for ensuring reliable RCS characterization under production line constraints.

To mitigate these undesired effects, a background subtraction technique is incorporated into the measurement workflow to suppress static and quasi-static scattering contributions introduced by the industrial surroundings. In practice, a background response is first recorded with the same system configuration but without the DUT, capturing the composite coupling and environmental clutter from nearby metallic structures, supports, and fixtures. During the subsequent DUT measurement, this previously recorded background is removed in post processing so that the remaining response is dominated by the scattering from the target within the quiet zone. To ensure that the subtraction remains valid, the robotic arm and conveyor are used only for transportation and positioning between tests, whereas the VNA data acquisition is triggered strictly after the DUT reaches the prescribed pose and becomes stationary. This stop-and-measure strategy minimizes time-varying multipath and motion-induced phase errors, improving the repeatability of the calibrated RCS extraction under streamline inspection conditions.

The RCS is obtained from the measured complex transmission coefficient $S_{21}$ using a vector network analyzer (VNA). Since the measured data include chamber coupling and fixture reflections, a background subtraction procedure was applied to isolate the target response:

(1) $$S_{21,DUT}(f)=S_{21,all}(f)-S_{21,bg}(f),$$

where $S_{21,all}(f)$ and $S_{21,bg}(f)$ denote the complex S-parameters with and without the target, respectively, under identical antenna configuration and VNA settings. The subtraction in (1) is performed in the complex domain.

After background subtraction, the absolute RCS of the DUT is determined by comparison with a standard calibration target, which is widely used in CATR calibration [23,28]. For convenience of explanation, the normalized RCS is utilized in this paper. All RCS values are normalized to the trihedral reference, expressed in dB.

In conventional TA-CATRs, the measurable angular range is restricted by feed positions and quiet zone uniformity (typically ±30°) [26]. To overcome this limitation, an angular extrapolation technique [27] is incorporated into the proposed system to extend the measurable RCS up to ±45°. The technique utilizes the beam deviation extrapolation model derived in [8]. Building on the observation that the measured coupling magnitude between the DUT and the TA-CATR feed exhibits a strong and regular dependence on the angular deviation within the main-beam region, a fitting assisted extrapolation strategy can be employed to extend the usable angular spectrum without dense mechanical scanning. In this strategy, a small set of representative angle samples is first acquired in a pre-calibration stage to establish a cosine-type fitting model, which captures the monotonic decay behavior around the beam peak and remains stable under a well-formed quiet-zone illumination. The fitted curve is then resampled at a finer angular interval to generate an augmented initial data set, which improves the numerical robustness for subsequent extrapolation. Finally, a spline-based extrapolation is applied to extend the diagnosis or reconstruction curve beyond the originally sampled angular range, enabling a significantly wider effective coverage while keeping the sampling effort minimal. By employing a fitting function combined with an extrapolation technique, the DUT RCS in the TA-CATR can be measured using a multi-probe, multi-position approach.

**Figure 2:**
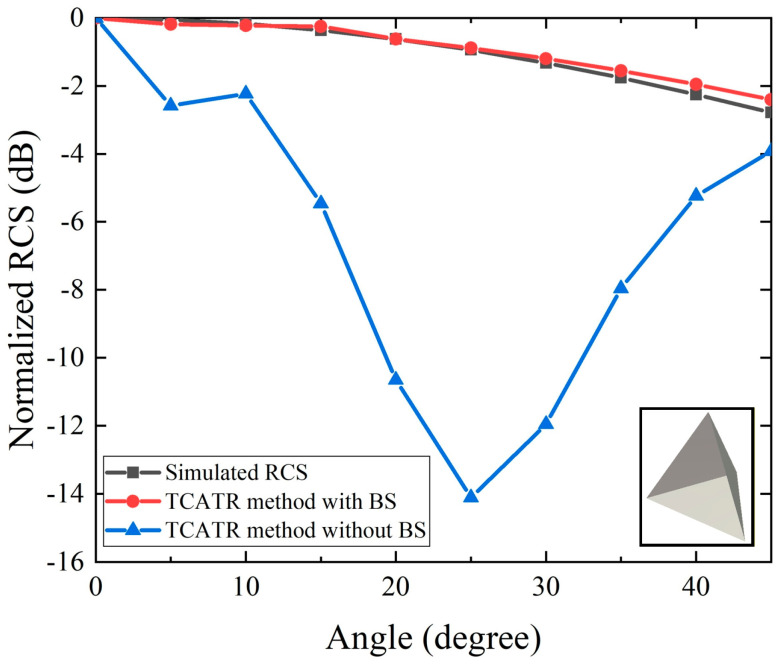
Comparison of simulated RCS versus elevation angle for the trihedral corner reflector.

In this study, a trihedral corner reflector with an edge length of 77.6 mm is selected as the DUT. The proposed TA-CATR provides a 13.5% fractional operational bandwidth centered at 2.6 GHz [12]. Considering the streamline measurement scenario and the plane wave illumination synthesized by the transmitarray within a compact near field region, the measured response is not solely determined by the target scattering. In practice, mutual coupling between the two probes, together with additional spatial scattering paths introduced by nearby metallic supports and surrounding fixtures, can superimpose an unwanted component on the received signal. This contamination becomes particularly evident when the DUT scattering is comparable to the parasitic background level, leading to a biased RCS estimate. This effect is reflected in Figure 2, where the raw curve shows abnormal magnitude deviations and reduced smoothness with respect to the expected angular trend, indicating contamination from quasi-static coupling and environmental clutter. To suppress these undesired contributions, a background subtraction method is incorporated into the proposed measurement framework. Specifically, the background response is first recorded under the same system configuration without the DUT, so that the composite coupling and static scattering from the environment are captured. The background term is then removed from the DUT measurement in post-processing, which largely eliminates the quasi-static interference and restores the dominance of the DUT scattering within the quiet zone. After this subtraction, the remaining samples follow a physically consistent angular dependence and can be further processed using the fitting and extrapolation procedures to compensate for the limited sampling range and to reconstruct the RCS magnitude over a wider angular coverage. As indicated by Figure 2, the proposed combination of background subtraction, fitting, and extrapolation effectively recovers the expected RCS magnitude trend and substantially improves the stability of the curve, enabling accurate RCS characterization in a compact and automation-oriented inspection environment. Consequently, the proposed TA-CATR enables wide angle (±45°) UAV/AAV RCS characterization in a compact and cost-effective configuration, well suited for automated streamline measurement. The extrapolation parameters are determined and validated using the canonical trihedral, so that the compensation mainly reflects the system illumination deviation rather than target-dependent scattering complexity.

**Figure 3:**
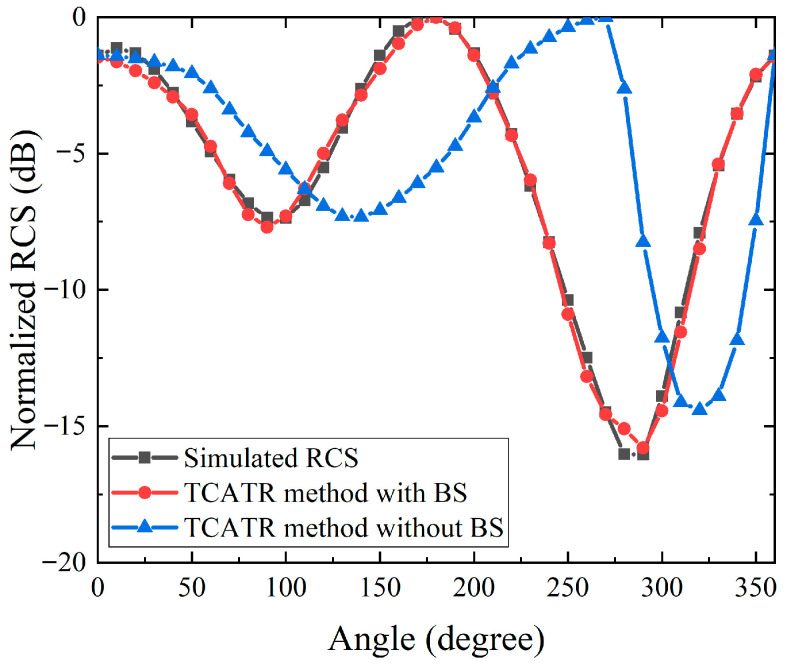
Comparison of simulated RCS versus azimuth angle for the trihedral corner reflector.

Furthermore, to characterize the RCS variation with azimuth angle in a streamline measurement setting, the DUT was rotated in azimuth and measured at sparse angular samples with a step of 45 degrees. For each azimuth state, the same background subtraction procedure was applied to suppress the quasi-static coupling and environmental scattering, ensuring that the extracted response is dominated by the DUT scattering under consistent illumination conditions. The discrete azimuth samples were then combined and interpolated to reconstruct a continuous azimuth-dependent RCS profile, as illustrated in Figure 3. The reconstructed curve follows the same overall trend as the reference full-wave RCS simulation, and the main lobes and null locations remain well aligned across the full 0 to 360 degree range. This close agreement in both magnitude level and angular variation verifies that the proposed measurement approach can reliably capture the azimuthal scattering signature with limited sampling effort, which is beneficial for streamline inspections.

**Figure 4:**
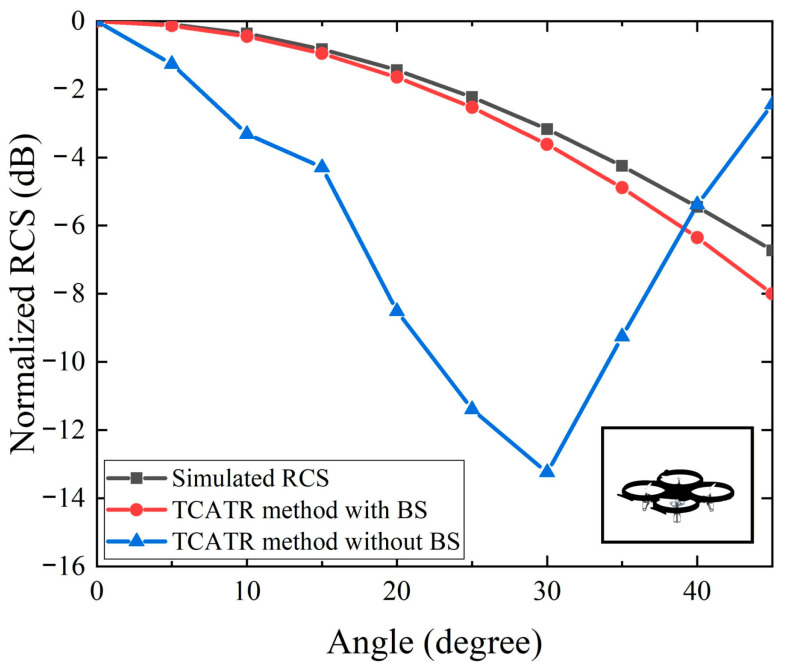
Comparison of simulated RCS versus elevation angle for the UAV/AAV.

**Figure 5:**
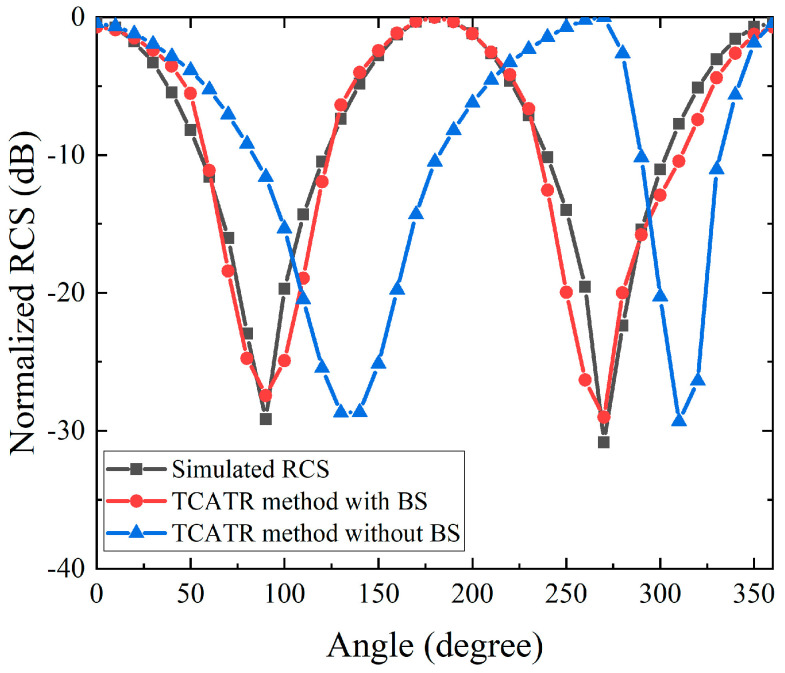
Comparison of simulated RCS versus azimuth angle for the UAV/AAV.

To further validate the effectiveness of the proposed testing methodology under realistic inspection conditions, a representative UAV or AAV structure with a maximum diameter of 100 mm was modeled as the DUT and evaluated in full-wave RCS simulations. Figure 4 and Figure 5 compare the elevation-angle and azimuth-angle RCS results obtained using the proposed processing chain against the reference simulation, respectively. For the elevation-angle case in Figure 4, the reconstructed RCS curve follows the same angular dependence as the reference data over the extended observation range, indicating that the proposed background subtraction, fitting, and extrapolation can effectively suppress industrial environment contamination while compensating for the systematic angular loss caused by the TA-CATR beam deviation beyond the nominal quiet-zone coverage. For the azimuth-angle case in Figure 5, the reconstructed profile also maintains good consistency with the reference simulation across 0 to 360 degrees, confirming that sparse sampling combined with interpolation can reliably capture the dominant scattering variation along the azimuth direction. Overall, the maximum deviation between the reconstructed results and the reference data is 1.3 dB, demonstrating that the proposed methodology achieves high accuracy and strong consistency for a non-canonical target geometry. The 1.3 dB maximum deviation represents an end-to-end discrepancy between the measurement and the simulation, and it includes system uncertainties such as VNA stability and alignment and positioning tolerances. The current results indicate that residual phase mismatch after background subtraction in the complex S21 domain is sufficiently small, and the fitting and extrapolation remain stable for trend level estimation. For more complex targets, accuracy can be improved by denser angular sampling and more robust fitting/averaging, together with periodic background refresh and reference monitoring.

It is noted that the extrapolation stage is primarily introduced to compensate for the systematic angular attenuation associated with the TA-CATR illumination deviation when the observation angle extends beyond the nominal quiet-zone coverage. Therefore, to isolate the compensation effect from target-dependent variability, the extrapolation procedure is first validated using a canonical trihedral reflector, whose scattering response is highly repeatable and exhibits a stable angular behavior. For more complex targets such as the UAV or AAV structure, the RCS may fluctuate rapidly with angle due to multiple scattering centers and geometry-dependent specular components. Under such conditions, the proposed method is intended to provide a trend-level wide-angle estimation suitable for streamlined inspection, rather than a fully resolved high-resolution angular signature. The effective angular resolution is mainly limited by the measured angular sampling, not the interpolated grid. Higher fidelity can be obtained by denser sampling, for example, 15 degrees or smaller, with the same processing workflow.

## 3. Experiment and Results

**Figure 6:**
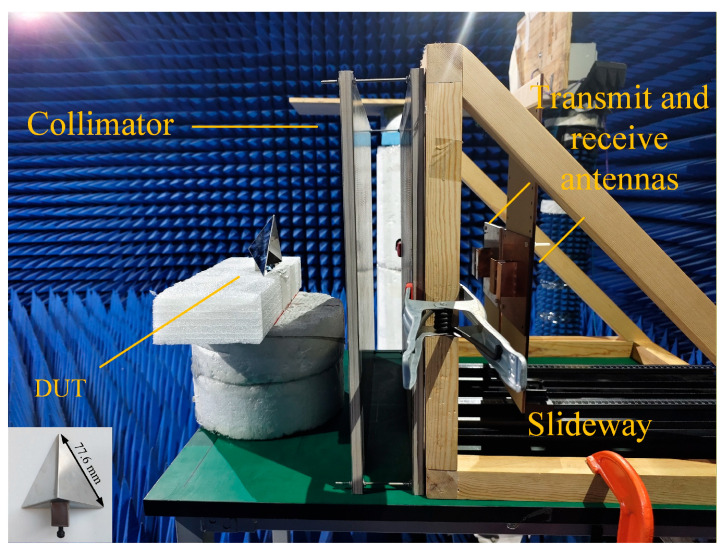
Proposed TA-CATR RCS measurement setup.

To experimentally validate the proposed methods, a dedicated measurement platform was established, as illustrated in Figure 6. The setup integrates the transmitarray-based compact antenna test range collimator, a supporting frame, a linear slide rail with a positioning bracket, a transmit antenna, a receive antenna, a DUT holder, and a vector network analyzer. The TA-CATR is used to generate a near plane wave illumination in the designated quiet zone, where the DUT is placed for RCS measurements. The transmit and receive antennas are mounted on the bracket and can be precisely translated along the linear slide rail, enabling accurate control of the antenna positions and ensuring repeatable alignment with respect to the TA-CATR focus and the quiet zone center. The DUT is fixed on the supporting structure in front of the quiet zone, and its rotation angle is calibrated using an angle gauge and a leveling instrument to reduce pose uncertainty during angular sampling.

All measurements are performed at a carrier frequency of 2.6 GHz under an *x*-polarized configuration. Although x-polarization is used here, the same processing can typically be applied to other polarization configurations after a consistent polarization calibration; differences mainly arise from the measurement setup. During the test, the VNA records the complex scattering response for each angular state following the same data acquisition and background subtraction procedure described in the methodology. As a verification example, a standard trihedral corner reflector is employed as a canonical target due to its stable and repeatable scattering behavior. This test object is used to demonstrate the complete measurement procedure and to validate the system performance before evaluating more complex DUT geometries.

**Figure 7:**
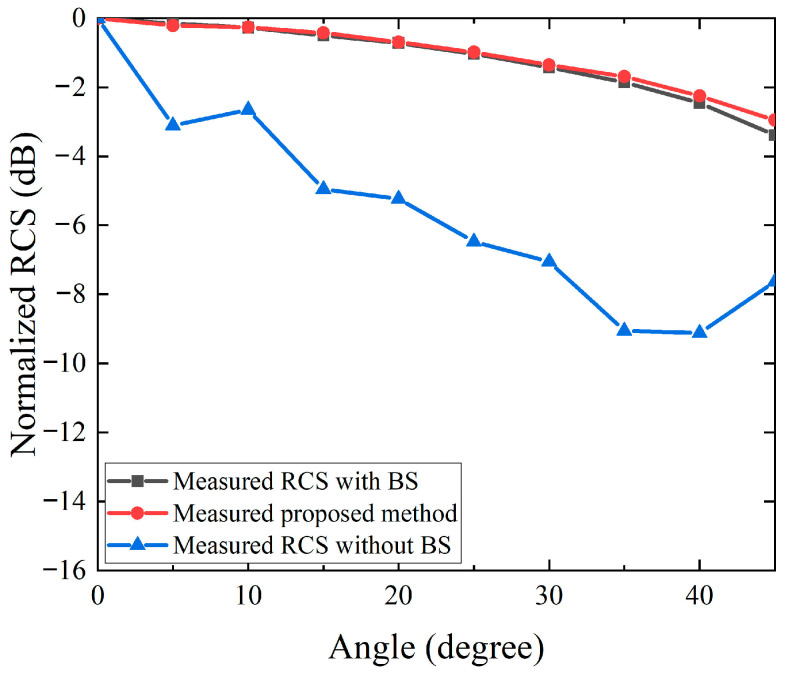
Comparison of measured RCS versus elevation angle for the trihedral corner reflector.

**Figure 8:**
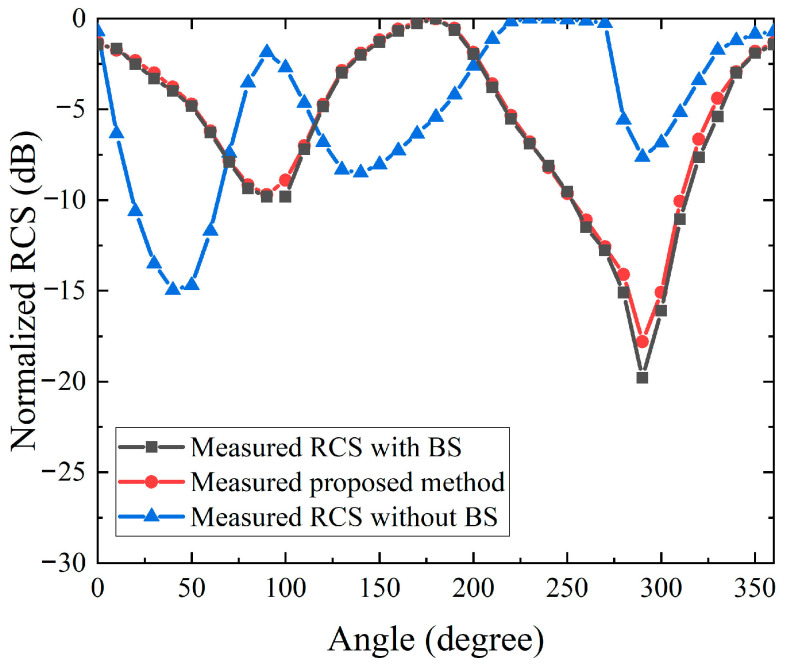
Comparison of measured RCS versus azimuth angle for the trihedral corner reflector.

Figure 7 and Figure 8 summarize the experimental validation results of the proposed RCS measurement method using the TA-CATR. Figure 7 focuses on the elevation angle characterization and highlights the impact of environmental clutter and probe coupling by showing the case without background subtraction. As can be observed, the curve obtained without subtraction deviates noticeably from the expected angular trend, indicating that quasi-static scattering contributions bias the elevation-dependent RCS estimation in the streamline test configuration. After applying the proposed processing chain, the background-corrected samples become physically consistent and can be accurately described by the fitting model around the main lobe region. On this basis, the extrapolation algorithm further compensates for the systematic angular attenuation beyond the nominal quiet zone coverage, thereby extending the effective measurement range to plus and minus 45 degrees. The reconstructed elevation angle curve remains smooth and follows the simulated reference data with good agreement over the extended angular span, demonstrating that the combined background subtraction, fitting, and extrapolation is effective for wide angle elevation characterization.

Figure 8 presents the azimuth angle measurements. To balance measurement efficiency and angular coverage, the RCS responses are sampled at sparse azimuth angles and processed with the same background subtraction procedure to suppress quasi-static clutter, after which interpolation is applied to reconstruct a continuous 0 to 360 degree RCS distribution. The reconstructed azimuth curve preserves the dominant variation features and maintains a high level of consistency with the measured reference data, confirming that the proposed sparse sampling and interpolation strategy is reliable for full azimuth evaluation. Overall, the experimental results in Figure 7 and Figure 8 verify that the proposed approach enables accurate and efficient bistatic RCS measurements using the TA-CATR, while remaining compatible with compact and automation inspection environments. A 45° angular step is used to reduce measurement time and simplify the test procedure for streamlined RCS characterization. The sampled data still show a clear angular trend and are well captured by the fitting model. For higher angular fidelity, a smaller step (e.g., 15°) can be used at the cost of longer measurement time.

During streamline measurement, the DUTs are transported by a conveyor belt and sequentially positioned within the quiet zone for inspection, where a stop-and-measure operation is executed to ensure data repeatability. In the elevation angle stage, the complex S-parameter responses of the DUT are first recorded using the predefined feed-antenna positions, which provides the essential samples for subsequent fitting and extrapolation while keeping the acquisition effort low. The DUT is then rotated in azimuth at 45 degree intervals, and the corresponding S-parameter data are collected at each orientation under the same polarization and measurement settings. In post-processing, the measured S-parameters are converted into calibrated RCS values as functions of elevation and azimuth angles by applying background subtraction to suppress quasi-static coupling and environmental clutter, followed by fitting and extrapolation for wide angle elevation reconstruction and interpolation for full azimuth reconstruction. Beyond RCS evaluation, the resulting wide angle scattering signatures can also serve as compact fingerprints for quality control. Similar to the approach described in [26], the same workflow can be extended to automated fault detection and structural verification for mass-produced UAVs and AAVs by comparing the reconstructed angular RCS profiles against reference templates and tracking deviations that indicate assembly errors, missing components, or mechanical deformation. Compared with conventional far-field ranges and bulky reflector-based CATRs, the proposed TA-CATR provides a more compact and cost-effective solution that is directly deployable on production lines, while still enabling accurate wide-angle and repeatable bistatic RCS measurements with coverage of plus and minus 45 degrees in elevation and 0 to 360 degrees in azimuth. Although demonstrated for batch UAV and AAV equipment, the streamline TA-CATR workflow is applicable to other targets that require compact, repeatable, and high-throughput RCS inspection. A trihedral reflector is used as the DUT because it provides a stable and repeatable reference response for validating the TA-CATR measurement chain and the proposed processing (background subtraction, angular extrapolation, and interpolation). For realistic UAVs with multiple scattering centers, the angular response may vary more rapidly and can exhibit oscillations due to scattering interference; thus, the extrapolation should be regarded as trend-level estimation within the sampled angular span, and higher fidelity can be obtained by denser angular sampling or piecewise fitting when needed. In production line deployment, the method is most reliable when the environment is quasi-static during each short measurement cycle; practical mitigation includes short acquisition windows, periodic background refresh, and reference target monitoring to detect environmental drift.

It is worth emphasizing that the proposed platform is designed with low cost and high efficiency as primary objectives for streamline inspection. The TA-CATR is fabricated using printed circuit board technology and relies on a compact mechanical fixture, which avoids bulky reflectors and large anechoic facilities and therefore reduces both hardware and installation cost. Moreover, the measurement workflow is tailored to high-throughput operation by combining background subtraction with sparse angular sampling. Only a small number of feed positions and azimuth states are required to reconstruct wide-angle RCS responses through fitting, extrapolation, and interpolation. As a result, the proposed approach provides an economical and practical pathway to achieve repeatable wide-angle RCS characterization in industrial environments. In contrast to Refs. [26,27], which focuses on peak beam angle calibration, this work targets scattering-based RCS characterization and therefore requires complex domain background subtraction before angular reconstruction. The system scales across frequencies and DUT sizes by redesigning the transmitarray aperture and feed to maintain quiet zone quality. Measurement efficiency can be improved via optimized angular sampling and automation, and multi-probe extensions can further reduce measurement time. The overall test time is mainly determined by the number of physical angular measurements, and the proposed sparse sampling workflow reduces this number while keeping the post-processing lightweight.

## 4. Conclusions

In this article, a rapid and low-cost RCS measurement framework is proposed and validated for streamline inspection of low-altitude unmanned communication equipment. A compact transmitarray compact antenna test range fabricated with printed circuit board technology is developed to provide near plane wave illumination within a limited near-field space. This enables practical deployment in industrial settings where conventional far-field ranges are difficult to implement. To improve robustness in open environments with metallic fixtures and unavoidable clutter, background subtraction is incorporated to suppress quasi-static probe coupling and environmental scattering. Based on the background-corrected data, fitting and extrapolation are employed to compensate for the systematic angular loss caused by illumination deviation outside the nominal quiet zone coverage, extending the effective elevation angle characterization to minus 45 degrees to plus 45 degrees. For azimuth angle evaluation, sparse sampling combined with interpolation reconstructs a continuous RCS distribution from 0 to 360 degrees with reduced acquisition effort. Experimental measurements using a standard trihedral corner reflector show close agreement with reference simulations, confirming the accuracy and repeatability of the proposed method. With its compact configuration, low cost, and high measurement efficiency, the proposed approach provides a practical and scalable solution for large-scale RCS inspection of UAV and AAV systems in future ISAC-oriented streamline measurement scenarios.

## Data Availability

All data are available from the author.

## References

[1] Potgieter M., Odendaal J.W., Blaauw C., Joubert J. (2019). Bistatic RCS measurements of large targets in a compact range. IEEE Trans. Antennas Propag..

[2] Ford K.L., Bennett J.C., Holtby D.G. (2013). Use of a plane-wave synthesis technique to obtain target RCS from near-field measurements, with selective feature extraction capability. IEEE Trans. Antennas Propag..

[3] Boga K.M., Kandimalla D., De A. (2026). Bistatic Radar Cross Section of Curved Plates Using Curved Screen Uniform Theory of Diffraction. IEEE Trans. Radar Syst..

[4] Piuzzi E., D’ATanasio P., Pisa S., Pittella E., Zambotti A. (2015). Complex radar cross section measurements of the human body for breath-activity monitoring applications. IEEE Trans. Instrum. Meas..

[5] Liu Y., Hu W., Zhang W., Sun J., Xing B., Ligthart L. (2020). Radar cross section near-field to far-field prediction for isotropic-point scattering target based on regression estimation. Sensors.

[6] Lee D.-Y., Lee J.-I., Seo D.-W. (2022). RCS estimation of drone motion using mesh-element rotation in MoM and micro-Doppler signatures. IEEE Trans. Antennas Propag..

[7] Xiao D., Guo L., Liu W., Hou M. (2021). Efficient RCS prediction of the conducting target based on physics-inspired machine learning and experimental design. IEEE Trans. Antennas Propag..

[8] Hu C., Li N., Chen W., Guo S. (2019). A near-field to far-field RCS measurement method for multiple-scattering target. IEEE Trans. Instrum. Meas..

[9] Zhang Y.X., Jiao Y.C., Zhu M.D., Zhang Y. (2023). A linear-scan-based wideband single-cut near-field RCS measurement technique with wide effective angle coverage. IEEE Antennas Wirel. Propag. Lett..

[10] Chen Y., Yu J., Ge H., Yao Y., Chen X. (2022). Unified initial preprocessing for phaseless characterization of quiet zone in millimeter-wave compact antenna test range. IEEE Antennas Wirel. Propag. Lett..

[11] Koskinen T., Ala-Laurinaho J., Saily J., Lonnqvist A., Hakli J., Mallat J., Tuovinen J., Raisanen A.V. (2005). Experimental study on a hologram-based compact antenna test range at 650 GHz. IEEE Trans. Microw. Theory Tech..

[12] Tang J., Chen X., Meng X., Wang Z., Ren Y., Pan C., Kishk A.A. (2022). Compact antenna test range using very small F/D transmitarray based on amplitude modification and phase modulation. IEEE Trans. Instrum. Meas..

[13] Lee T.-H., Burnside W.D. (1997). Compact range reflector edge treatment impact on antenna and scattering measurements. IEEE Trans. Antennas Propag..

[14] Chen T., Chen X., Yao Y., Zhang L., Liu X., Yu J. (2023). Theoretical verification of single-reflector compact antenna test range with high aperture usage. IEEE Trans. Antennas Propag..

[15] Multari M., Lanteri J., Le Sonn J.L., Brochier L., Pichot C., Migliaccio C., Desvilles J.L., Feil P. (2010). 77 GHz stepped lens with sectorial radiation pattern as primary feed of a lens based CATR. IEEE Trans. Antennas Propag..

[16] Zatta R., Jagtap V.S., Grzyb J., Pfeiffer U.R. (2021). Broadband lens-integrated CMOS camera-type THz compact antenna test range. IEEE Trans. THz Sci. Technol..

[17] Yang K., Wang Z., Chen X., Loh T.H., Gao S. (2025). Enhancing aperture efficiency of single parabolic cylindrical compact range based on a linear array excitation method. IEEE Trans. Antennas Propag..

[18] Vaquero Á.F., Arrebola M., Pino M.R., Florencio R., Encinar J.A. (2021). Demonstration of a reflectarray with near-field amplitude and phase constraints as compact antenna test range probe for 5G new radio devices. IEEE Trans. Antennas Propag..

[19] Li Z., Huo P., Wu Y., Wu J. (2021). Reflectarray compact antenna test range with controlled aperture disturbance fields. IEEE Antennas Wirel. Propag. Lett..

[20] Liu F., Cui Y., Masouros C., Xu J., Han T.X., Eldar Y.C., Buzzi S. (2022). Integrated sensing and communications: Toward dual-functional wireless networks for 6G and beyond. IEEE J. Sel. Areas Commun..

[21] Ghazzai H., Menouar H., Kadri A., Massoud Y. (2019). Future UAV-Based ITS: A Comprehensive Scheduling Framework. IEEE Access.

[22] Ghazzai H., Ghorbel M.B., Kadri A., Hossain M.J., Menouar H. (2017). Energy-efficient management of unmanned aerial vehicles for underlay cognitive radio systems. IEEE Trans. Green Commun. Netw..

[23] Yuan Z., Yu L., Wang Z., Li C., Dallmann T., Fan W. (2025). Experimental analysis and modeling of monostatic AAV RCS for ISAC channels. IEEE Antennas Wirel. Propag. Lett..

[24] Ghazzai H., Kadri A., Ghorbel M.B., Menouar H., Massoud Y. (2018). A generic spatiotemporal UAV scheduling framework for multi-event applications. IEEE Access.

[25] Ezuma M., Anjinappa C.K., Funderburk M., Guvenc I. (2022). Radar cross section based statistical recognition of UAVs at microwave frequencies. IEEE Trans. Aerosp. Electron. Syst..

[26] Tang J., Meng X., Chen X., Chen R., Da Y., Zhu S., Zhang A., Yu M., Kishk A.A. (2023). Efficient angle calibration method for peak beam measurements in transmitarray-based compact antenna test range. IEEE Trans. Electromagn. Compat..

[27] Tang J., Huang G.L., Zhang A., Chen X. (2024). Enhanced peak beam deviation estimation based on extrapolation method for a transmitarray compact antenna test range. IEEE Antennas Wirel. Propag. Lett..

[28] Jarvis R.E., Mattingly R.G., McDaniel J.W. (2021). UHF-band radar cross section measurements with single-antenna reflection coefficient results. IEEE Trans. Instrum. Meas..

